# Green Extraction of Phenolic Compounds from Lotus Seedpod (*Receptaculum Nelumbinis*) Assisted by Ultrasound Coupled with Glycerol

**DOI:** 10.3390/foods10020239

**Published:** 2021-01-25

**Authors:** Nina Bao, Di Wang, Xizhe Fu, Hujun Xie, Guizhen Gao, Zisheng Luo

**Affiliations:** 1 Suzhou Engineering and Technological Research Center of Natural Medicine and Functional Food, School of Biological and Food Engineering, Suzhou University, Suzhou 234000, Anhui, China; ninabao@ahszu.edu.cn; 2 Zhejiang Key Laboratory for Agro-Food Processing, National-Local Joint Engineering Laboratory of Intelligent Food Technology and Equipment, College of Biosystems Engineering and Food Science, Zhejiang University, Hangzhou 310058, Zhejiang, China; wangdi237@zju.edu.cn (D.W.); xizhefu@zju.edu.cn (X.F.); 3 School of Food Science and Biotechnology, Zhejiang Gongshang University, Hangzhou 310058, Zhejiang, China; hujunxie@gmail.com; 4 Ningbo Research Institute, Zhejiang University, Ningbo 315000, Zhejiang, China; 5 Fuli Institute of Food Science, Hangzhou 310058, Zhejiang, China

**Keywords:** *Receptaculum Nelumbinis*, phenolic compound, ultrasonic-assisted extraction, antioxidant activity, Fourier transform infrared (FTIR), Scanning electron microscopy (SEM)

## Abstract

Lotus *Receptaculum Nelumbinis* has been sparking wide research interests due to its rich phenolic compounds. In the present work, ultrasonic-assisted extraction coupled with glycerol was employed to extract phenolic compounds from *Receptaculum Nelumbinis* and the process was optimized using a response surface methodology with Box-Behnken design (BBD). The optimal conditions for the total phenolic content (TPC) extract were obtained: glycerol concentration of 40%, an extraction temperature of 66 °C, ultrasonic time of 44 min, and the solvent-to-solid ratio of 55 mL/g. Under these optimum extraction conditions, the extraction yield of TPC was 92.84 ± 2.13 mg gallic acid equivalents (GAE) /g. Besides, the antioxidant activities demonstrated the ability of free radical scavenging by four different methods that included 2,2-Diphenyl-1-picrylhydrazyl (DPPH), ferric reducing antioxidant power (FRAP), 2,2′-azinobis(3-ethylbenzothiazoline-6-sulfonic acid) (ABTS), and reducing activity (RA) were 459.73 ± 7.07, 529.97 ± 7.30, 907.61 ± 20.28, and 983.66 ± 11.80 μmol TE/g, respectively. Six phenolic compounds were identified by ultra-high pressure liquid chromatography combined with triple-time-of-flight mass spectrophotometry (UPLC-Triple-TOF/MS) from the extracts. Meanwhile, Fourier transform infrared (FTIR) was conducted to identify the characteristic functional groups of the extracts and thus reflected the presence of polyphenols and flavonoids. Scanning electron microscopy (SEM) illustrated the microstructure difference of four treatments, which might explain the relationships between antioxidant activities and the structures of phenolic compounds.

## 1. Introduction

Lotus (*Nelumbo nucifera Gaertn.*) is a perennial freshwater plant widely cultivated in Asia and most parts are edible, so in China, they have been used for pharmaceutical purpose [1,2,3,4]. Lotus seedpod (LSP) is one of the main byproducts of lotus, which is discarded directly and leads to a large quantity of waste during the lotus seed processing [5]. However, LSP has been reported to be rich in phenolic compounds, i.e., gallic acid, catechin, caffeic acid, quercetin, kaempferol, and possesses various physiological activities, such as antioxidant, antimicrobial, and anti-inflammatory, which make it interesting for incorporation as an important and valuable ingredient into the medicine, food, and cosmetics industry [6]. Therefore, the extraction of phenolic compounds from different types of by-products in the food industry can attach more value to LSP and meets the concept of an eco-friendly economy [7].

The approach for extraction is a critical procedure for the phenolic compound recovery, which has attracted much attention [8]. Traditional methods including refluxing, soxhlet extraction, maceration, and boiling possessed many disadvantages such as low extraction efficiency, time-consuming, energy-consuming, and high toxicity of the solvent [9]. Therefore, many innovative techniques have gained the interest of researchers in this area over the past years involving ultrasonic-assisted extraction (UAE), microwave-assisted extraction, solid-phase extraction, accelerated solvent extraction, and pressurized liquid extraction [10]. Among these techniques, UAE has been considered to be one of the most promising techniques due to its high efficiency, easy-handling, energy-saving and eco-friendliness, and easiness to scale-up for industry [11,12].

Additionally, traditional organic solvents, such as methanol, ethanol, and acetone were widely used during the extraction process, for the reason of their dissolving capacity and efficiency. However, the defects of these organic solvents including potential environmentally hazardous, unacceptable solvent residues in the extracts were increasingly competing with the environmental concern of the public [13]. Therefore, green solvents, such as glycerol [14] and polyethylene glycol [15] were considered as green and cheap alternatives to the traditional harmful organic solvents.

The aims of this study were: (1) to investigate the effect of UAE process variables on the yield of total phenolic compounds by using response surface methodology (RSM) and subsequently obtain the optimal extraction conditions; (2) to explore the in vitro antioxidant activities of the extracts with four different methods including DPPH assay, FRAP assay, ABTS assay, and RA assay; (3) to illustrate the difference of functional groups in the extracts from LSP and microstructure of LSP among different extraction strategies by using Fourier transform infrared (FTIR) and Scanning electron microscopy (SEM).

## 2. Materials and Methods

### 2.1. Plant Material and Reagents

The dried LSP discarded during processing were collected from Hangzhou, Zhejiang province, China, and dried with hot air at 45 °C until reaching a constant weight. Folin-Ciocalteu phenol reagent, quercetin, vanillin, and glycerol were purchased from Macklin, gallic acid, DPPH, ABTS, 2,4,6-tripyridyl-s-triazine (TPTZ), ferric chloride, sodium carbonate (Na_2_CO_3_), sodium nitrite (NaNO_2)_, aluminum chloride (AlCl_3_), sodium hydroxide (NaOH), methanol, hydrochloric acid, catechin, Folin-Denis reagent, tannin acid, Trolox, phosphate, potassium ferricyanide, trichloroacetic acid, ferric chloride, and L-ascorbic acid were purchased from Aladdin Industrial Co. (Shanghai, China). All solvents of HPLC grade were purchased from Aladdin Industrial Co., Ltd. (Shanghai, China).

### 2.2. Sample Preparation

The dried LSP was ground to a powder with a pulverizer (Huangcheng, HC-280T, Jinhua, Zhejiang, China) and is passed through a 40-mesh sieve and stored at −20 °C before analysis. A 400 W ultrasonic cleaning bath (Shengxi, DS-8510DTH, Shanghai, China) was employed for the extraction process of the LSP at a frequency of 40 KHz. An external water bath system was used. The powder of LSP (0.25 g) was placed into a 50 mL plastic centrifuge tube, and an amount of glycerol was added and sonicated at different temperatures for varying time periods. Subsequently, the mixture was centrifuged at 10,950× *g* for 15 min (Bioridge, TGL-16M, Shanghai, China) and the supernatant was obtained for further analysis.

### 2.3. RSM Design

The concentration of glycerol, ultrasonic power, ultrasonic time, solvent-to-solid ratio, and extraction temperature were tested under different conditions by single-factor experiments. After selecting the optimal level of each factor based on the highest extraction yield of TPC, the RSM with a four-factor-three-level BBD was applied to investigate the influence of four independent variables: concentration of glycerol (X_1_), ultrasonic time (X_2_), temperature (X_3_), and solvent-to-solid ratio (X_4_). A total of 29 experimental runs were conducted for the RSM. The process variables and their code variable levels are shown in Table 1. The experimental data were described using a second-order polynomial model equation to obtain the regression coefficients. The 3D graphical analysis was also carried out by using Design-Expert software (8.0.6). The analysis of variance (ANOVA) was carried out to evaluate the individual linear, quadratic, and interaction terms. *F*-value, lack of fitness, and coefficient of determination (*R*^2^) were calculated for the fitness of the polynomial equation of each response.

### 2.4. Phenolic Compounds Analysis

The TPC of the extracts was determined according to previous work by Limwachiranon et al. [16]. TPC was expressed as mg of GAE per gram of dried LSP (mg GAE/g).

The total flavonoid content (TFC) of the extracts was determined according to Li et al. [17] with slight modifications. In brief, 750 μL samples were mixed with 45 μL 5% NaNO_2_ solution and incubated for 5 min at room temperature in the dark. Then, 45 μL of AlCl_3_ solution (10%) was added to react for another 5 min. After that, 300 μL 1 mol/L NaOH solution was added to stop the reaction and 360 μL distilled water was added to measure the absorbance at 510 nm (Tecan, Spark, Shanghai, China). TFC was expressed as mg of quercetin equivalent (QE)/g of dried LSP.

Total condensed tannins content (TCTC) of the extracts was determined according to Koutsoukos et al. [18], with slight modifications. Briefly, 150 μL samples were mixed with 1000 μL vanillin methanol solution and 500 μL hydrochloric acid. After 15 min of incubation at room temperature and protection from the light, the absorbance was measured at 500 nm (Tecan, Spark, Shanghai, China). TCTC was expressed as mg of catechin equivalent (CE)/g of dried LSP.

Total tannins content (TTC) of the extracts was determined by the Folin-Denis colorimetric method according to Swain et al. [19] with slight modifications. In brief, 1 mL samples were added into a 50 mL brown glass volumetric flask, mixed with 2.5 mL Folin-Denis reagent and 5 mL saturated sodium carbonate solution, then distilled water was added up to 50 mL. After 30 min of incubation under room temperature and protection from the light, the absorbance was measured at 760 nm (Tecan, Spark, Shanghai, China). TTC was expressed as mg of tannic acid equivalent (TAE)/g to dried LSP.

### 2.5. Antioxidant Activities

Antioxidant activity in terms of the DPPH assay was determined as reported by Brand-williams [20], with slight adjustments. In brief, 2 mL samples were added with 4 mL 0.2 mmol/L DPPH reagent, the mixture was vortexed and then incubated for 30 min in the dark at room temperature. The absorbance was measured at 517 nm (Metash, UV-5800PC, Shanghai, China) and expressed as mg of Trolox equivalent (TE)/g to dried LSP.

The FRAP assay was determined according to Oldoni et al. [21], with slight modifications. Briefly, Trolox was used for calibration and results were expressed as mg of Trolox equivalent (TE)/g to dried LSP.

The ABTS ^+^ assay was determined as reported by Marmouzi et al. [22].

The RA assay was determined as reported by Oyaizu [23], with slight modifications. In brief, 200 μL samples were mixed with 0.5 mL phosphate buffer (200 mmol/L, pH = 6.6) and 0.5 mL potassium ferricyanide solution (1.0%, *w*/*v*), the mixture was vortexed and then incubated for 30 min at 50 °C. After that, 0.5 mL trichloroacetic acid solution (10.0%, *w*/*v*) was added to the mixture and then centrifuged at 2500× *g* for 10 min. Then 1.2 mL supernatant was decanted to another tube and mixed with 0.12 mL ferric chloride solution (0.1%, *w*/*v*). The absorbance was measured at 700 nm (Metash, UV-5800PC, Shanghai, China).

### 2.6. Identification of Constituents by UPLC-Triple-TOF/MS

The ultra-high pressure liquid chromatography combined with triple-time-of-flight mass spectrophotometry (UPLC-Triple-TOF/MS) analysis was conducted according to Zeng et al. [24]. Waters UPLC (Waters Corp., Milford, MA, USA) was coupled with the AB Triple TOF 5600 plus System (AB SCIEX, Framingham, MA, USA) for analysis. ACQUITY UPLC HSS T3 column (Waters Corp., 1.7 μm, 3.0 × 50 mm, Milford, MA, USA) was used in all the chromatographic experiments. The mobile phases were 0.1% aqueous formic acid solution (A) and 0.1% formic acid-acetonitrile (B). The linear gradient programs were 0/5, 12/30, and 15/95 (min/B%); sample injection volume, 10 μL; column oven temperature, 35 °C; flow rate, 0.5 mL min^−1^; the UV detector was set at 280 nm. The optimal MS conditions: scan range *m*/*z* 100-2000. Negative ion mode: source voltage was −4.5 kV and the source temperature was 550 °C. The pressure of gas 1 (air) and gas 2 (air) was set to 50 psi and the pressure of the curtain gas (N_2_) was set to 35 psi. The maximum allowed error was set to ± 5 ppm.

### 2.7. FTIR Spectrometry Analysis

All treatments of LSP extracts using different methods were characterized by FTIR spectrometer (Thermo Scientific, Nicolet iS50 Spectrometer, Waltham, MA, USA) equipped with OMNIC software to identify the different characteristics of peak values and their active functional group. KBr was used to make the film, FTIR spectra were acquired in the wave numbers range of 4000–400 cm^−1^, and 32 scans were taken as spectra. Meanwhile, single spectra were corrected against the background spectrum of air.

### 2.8. Scanning Electron Microscope (SEM)

Microscopic analysis was done as reported by Zhou et al. [13] (Zhou et al., 2018). The dried LSP samples were mounted on aluminum stubs and coated with gold-palladium, and then were observed using a Gemini SEM 300 scanning electron microscope (Carl Zeiss, Oberkochen, Baden-Württemberg, Germany).

### 2.9. Statistical Analysis

Statistical analysis was performed using the IBM SPSS 20 software; one-way analysis of variance (ANOVA) and Tukey’s test was used for statistical analysis (*p* < 0.05 was considered as significant). All experiments were performed in triplicate and the data were reported as means ± standard deviation (SD).

## 3. Results and Discussion

### 3.1. Single Factor Analysis

**Figure 1:**
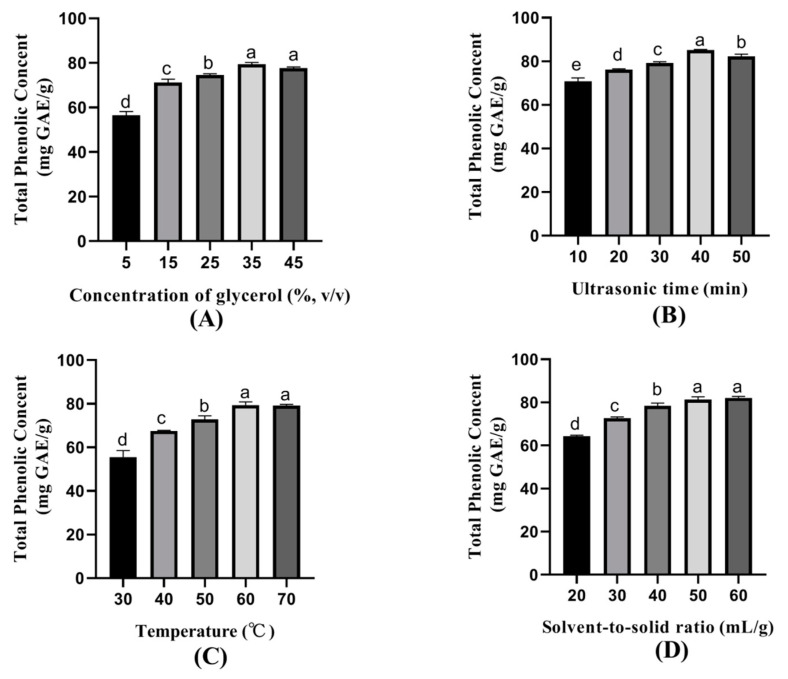
Effect of (**A**) concentration of glycerol, (**B**) ultrasonic time, (**C**) temperature, (**D**) solvent-to-solid ratio on total phenolic content. Data are reported as mean ± SD (*n* = 3). Different letters, such as a, b, c, d, and e, showed significant difference (*p* < 0.05).

The influence of single factors on the extracted amounts of phenolic compounds was studied, namely concentration of glycerol, ultrasonic time, temperature, solvent-to-solid ratio, and ultrasonic power (data not shown) (Figure 1). The glycerol concentration of 5–45% was chosen to evaluate the effect on the extraction yield of TPC (Figure 1A); 25–45% glycerol extracted more TPC, especially for 35% glycerol. Therefore, 35% of glycerol was thought to be the optimum concentration. The ultrasonic time ranging from 10 min to 60 min was screened to evaluate its influence on the extraction yield of TPC (Figure 1B). TPC extraction showed a relatively high yield with the ultrasonic time of 30–50 min, and 40 min showed the highest TPC extraction yield, which was considered as the optimum time.

Temperatures ranging from 30–70 °C were selected to estimate the influence on the extraction yield of TPC (Figure 1C). Temperatures of 60–70 °C showed a higher TPC extraction yield. Although the extraction yield of 60 °C was slightly higher than that of 70 °C, the low temperature indicated a low requirement for energy consumption. Thus 60 °C was considered as the optimum temperature. Solvent-to-solid ratios of 20–60 mL/g were selected to assess the impact on TPC extraction yield (Figure 1D). The TPC extraction presented a relatively high yield with the solvent-to-solid ratios of 50–60 mL/g. A low solvent-to-solid ratio means low solvent cost and high efficiency. Therefore, the solvent-to-solid ratios of 50 mL/g were selected.

### 3.2. Fitting the Model

The BBD was used to study the influence of extraction variables on TPC based on the results of preliminary experiments. A total of 29 experiments obtained with BBD for the response of TPC are shown in Table 2. ANOVA was applied to analyze the significance of the coefficients of experimental models and the accuracy of the model (Table 3). As shown in Table 3, *F*-value, *p*-value (Prob > *F*), and lack of fit value for dependent variables were 346.68, <0.0001 (remarkably significant), and 0.3679 (not significant), respectively. These findings indicated that the model could adequately fit the experimental real point to explain the response results. The coefficients of determination (*R*^2^) and adjusted *R*^2^ values were 0.9860 and 0.9942, respectively, which indicated that the predicted values from the model were close to the observed experimental results. The fitted second-order regression equations to predict TPC extraction yield was obtained by Equation (1).

TPC = 89.19 + 3.13X_1_ + 1.74X_2_ + 5.08X_3_ + 1.68X_4_ + 1.17X_1_X_2_ − 0.11X_1_X_3_ + 0.80X_1_X_4_ + 0.43X_2_X_3_ + 0.64X_2_X_4_ + 0.76X_3_X_4_ − 4.09X_1_^2^ − 3.61X_2_^2^ − 4.36X_3_^2^ − 2.83X_4_^2^ (1)

**Figure 2:**
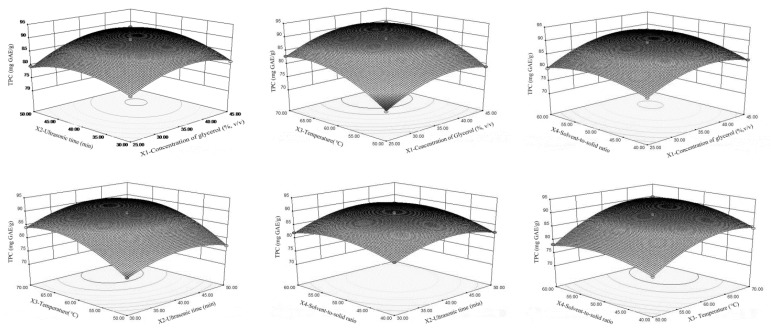
Response surface plots for the effect of test parameters on total phenolic content (TPC).

As shown in Table 3, all of the linear effects namely X_1_, X_2_, X_3_, X_4_, and their quadratic showed a remarkably significant (<0.0001) effect on TPC. Figure 2 showed the relationship between the TPC and extraction parameters. The interactions between the concentration of glycerol and ultrasonic time (X_1_X_2_) (<0.0001), the concentration of glycerol and solvent-to-solid ratio (X_1_X_4_) (0.0008), ultrasonic time and extraction temperature (X_2_X_3_) (0.0426), ultrasonic time and solvent-to-solid ratio (X_2_X_4_) (0.0044), and extraction temperature and solvent-to-solid ratio (X_3_X_4_) (0.0013) showed a positive effect on the extraction yield of TPC. The increase of glycerol could affect the solvent polarity [25] or change the dielectric constant of aqueous solution [26], which enhanced the solubility of TPC and subsequently increased the extraction efficiency of polar polyphenols. The mechanism of polyphenols solubility in different glycerol concentrations is complicated, due to various plant materials and other parameters, such as the intermolecular forces between the plant materials and the glycerol [27] as well as the external forces from ultrasonic. Our findings suggested that the optimal concentration of glycerol for TPC extraction from LSP by UAE was 40%. However, previous research demonstrated the optimum concentration of glycerol using a shaking water bath method was 85% [14]. Generally, prolonging the ultrasonic time could completely rupture the plant cells, assisting to disperse the solvent and dissolve phenolic compounds, subsequently improving the extraction efficiency. Besides, the prolonged ultrasonic time usually led to the thermal degradation of phenolic compounds due to light or oxygen exposure [28] or the promotion of oxidative degradation by acoustic cavitation from ultrasound [29]. Extraction temperature affects the solubility of TPC, the viscosity of the solvent, and the surface tension. Therefore, with the enhancement of extraction temperature, the TPC extraction yield is increased [30]. However, extremely high temperatures could lead to the possible degradation of the thermal-sensitive phenolic compounds [31] and might as well decrease the cavitation intensity and increase the vapor pressure. Thus, the ultrasound-assisted extraction process is affected [32]. For solvent-to-solid ratio, more solvent can promote more TPC to permeate into the solvent, leading to the increase in the extraction yield of TPC. However, as the solvent-to-solid ratio continues to increase, TPC begins to decrease slightly, because the viscosity of glycerol may affect the mass transfer [26].

### 3.3. Validation of the Optimum Conditions

Based on the RSM results, the optimal conditions were as follows: concentration of glycerol of 39.78% (adjusted to 40%), extraction temperature of 66.39 °C (adjusted to 66 °C), ultrasonic time of 44 min, and 54.96 mL/g (adjusted to 55 mL/g) as the optimum solvent-to-solid ratio. Under these conditions, the predicted TPC was 92.34 mg GAE/g. After performing the verification treatment, the TPC was at 92.84 ± 2.13 mg GAE/g, which correlates well with the model prediction.

### 3.4. Extraction Yield of TFC, TCTC, and TTC

Table 4 presented the comparative analysis of ultrasonic coupled with glycerol (UG) and other commonly used extraction methods, such as ultrasonic using water (UW), water bath incubation with glycerol (WG), and water bath incubation using water (WW) on the extraction yield of TFC, TCTC, and TTC under the optimal conditions. As for TFC, UG and UW showed much higher extraction yields than WG and WW. This phenomenon could be attributed to the cavitation which helps to disrupt plant cell walls and release amounts of TFC into the solvent [33,34,35]. As for TCTC, UG and WG showed a remarkably higher extraction yield than UW and WW, because the polarity of glycerol led to the dissolving of TCTC in the solvent. The results indicated that glycerol could change the dielectric constant of water and modified the polarity of the medium, which played a critical role in the extraction yield of TCTC [36,37]. However, the cavitation from ultrasound played a little role in it. Treatment of WG showed the highest extraction yield on TTC due to the polarity of glycerol, which was consistent with a previous work [38]. Besides, TTC by UG extraction was much lower than WG due to the loss of ultrasonication post-processing [39].

### 3.5. Antioxidant Activities

**Figure 3:**
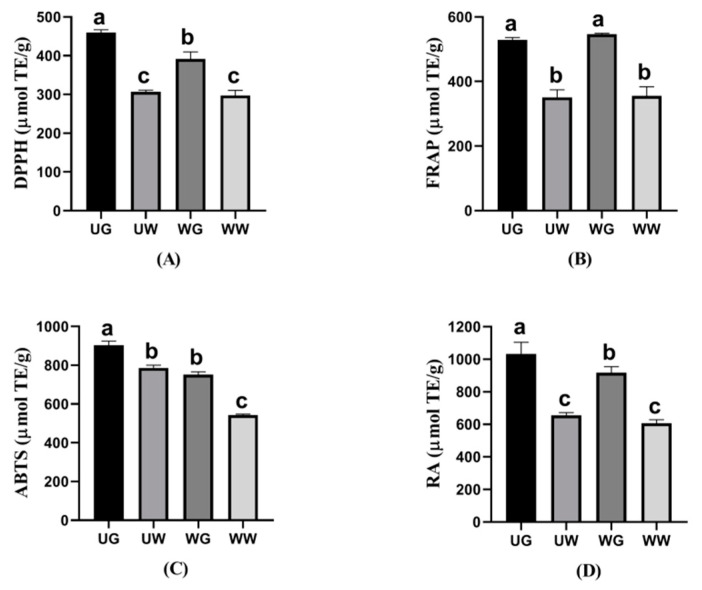
Antioxidant activities (**A**) DPPH, (**B**) FRAP, (**C**) ABTS, and (**D**) RA of TPC with four different treatments. Data are reported as mean ± SD (*n* = 3). Different letters, such as a, b, and c, showed significant difference (*p* < 0.05).

To assess the antioxidant activities of TPC from LPS, four in vitro antioxidant indexes by different antioxidant mechanisms were used, such as the DPPH, FRAP, ABTS, and RA assays. According to Figure 3, the results of antioxidant activities showed good antioxidant ability which was consistent with previous research [6]. UG had an obviously higher antioxidant capacity than UW, WG, and WW through DPPH, ABTS, and RA assays, which was due to the cavitation effects’ promoting the mass transfer of TPC to glycerol solutions. Meanwhile, previous studies revealed that the phenolic compounds and the sub-groups of phenolic compounds, such as TFC, TCTC, and TTC, could affect the antioxidant capacity [40]. In the present work, the highest antioxidant capacities through DPPH, ABTS, and RA assays did also coincide with higher TFC, TCTC, and TTC obtained by UG, which could explain the correlation between the antioxidant activities with polyphenol classes. Furthermore, WG showed non-significant higher antioxidant power on FRAP than UG, which may be due to the highest TTC extracted by the WG method. That is, TTC as a group of polyphenols possesses great radical scavenging activity and could increase the antioxidant capacity, especially for FRAP. It could be inferred that TTC would probably be t responsible for LPS’ FRAP antioxidant activity. WW showed the lowest antioxidant capacities throughout the four in vitro antioxidant assays, which suggested the lowest efficiency of water soaking among all the tested approaches during the extracting of TPC and sub-groups of phenolic compounds.

### 3.6. Identification of Constituents by UPLC-Triple-TOF/MS

Based on the MS and MS/MS spectral data compared with the data from the literature [14,41,42], six phenolic compounds, i.e., gallocatechin, gatechin, myricetin 3-*O*-glucuronide, isoquercetin, quercetin 3-*O*-glucuronide, and kaempferol 3-*O*-glucuronide were identified by UPLC-Triple-TOF/MS from the UG extract at optimum conditions. Among the phenolic compounds, the predominant compound was found to be quercetin 3-*O*-glucuronide, and a similar result was also reported by Huang et al. [14]. Furthermore, all of the six phenolic compounds have been tentatively identified in LPS extracts by recent detailed investigations [14]. Interestingly, four compounds, such as catechin, isoquercetin, quercetin 3-*O*-glucuronide, and kaempferol 3-*O*-glucuronide were also identified by Yan et al. [6], however, gallocatechin and myricetin 3-*O*-glucuronide were not reported by Yan et al. This is most probably due to the solvents used on the extraction of LPS with different polarity, which also was described by Apostolakis et al. with the phenomenon on solvent polarity [41].

### 3.7. FTIR Analysis

**Figure 4:**
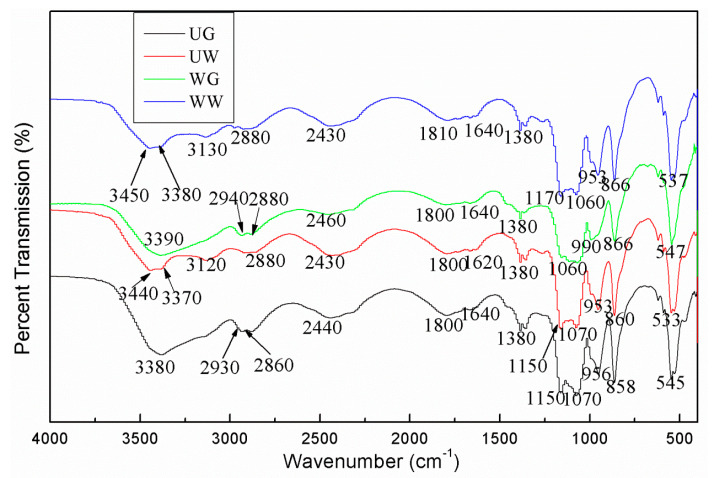
FTIR of LSP extract with four different treatments.

The FTIR analysis was conducted to identify and elucidate the functional groups in the plant extract samples. The FTIR spectra of the extracts from LSP at the optimized conditions by four different extract treatments are illustrated in Figure 4, which could validate the presence of phenolic compounds in the extracts. A broad absorption band at 3370 cm^−1^, 3380 cm^−1^, 3390 cm^−1^, 3440 cm^−1^, and 3450 cm^−1^ can be found, corresponding to the stretching vibration of hydroxyl groups or the presence of phenolic compounds (OH wagging) in LSP extracts [42,43,44,45,46,47]. The double peaks of UW and WW bands at 3450 cm^−1^ and 3370 cm^−1^ could be attributed to water. The bands at 2860 cm^−1^, 2880 cm^−1^, 2930 cm^−1^, and 2940 cm^−1^ were ascribed to the C-H stretching vibration of methyl groups, suggesting the presence of lipid-carbohydrate (CH_2_ and CH_3_ groups) [43,44,45,48]. The double peaks of UG and WG, with bands at 2940 cm^−1^ and 2860 cm^−1^, could be attributed to glycerol. The spectra at 1800 cm^−1^ and 1810 cm^−1^ reflected the stretching vibration of the carbonyl group (C=O), which was also observed in a previous work [47]. The peaks at 1620 cm^−1^ and 1640 cm^−1^ indicated the presence of C=O stretching [49] or the presence of aromatic ring deformations, or the presence of C=C stretching vibration [46], indicating the presence of polyphenols and flavonoids [43]. The peak at 1380 cm^−1^ indicated the presence of tannins, flavonoids, and glycosides [42]. The bands in the range of 1060–1170 cm^−1^ indicated the presence of flavonoid [45]. The peaks at 858-866 cm^−1^ indicated the presence of stretching vibrations of CH and CH_2_ [48]. Therefore, the characteristic functional groups reflected the presence of polyphenols and flavonoids, which were correlated with the results of UPLC-Triple-TOF/MS.

### 3.8. SEM

**Figure 5:**
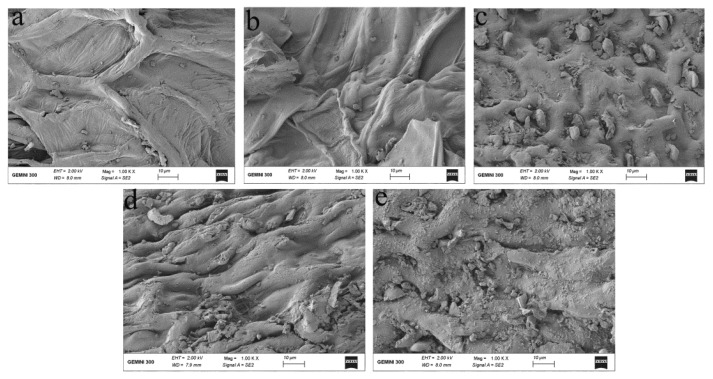
Scanning electron micrographs of LSP before (**a**) and after extraction with four different treatments (WW (**b**), WG (**c**), UW (**d**), and UG (**e**)).

As shown in Figure 5, the microstructures of LSP before and after extractions with various treatments were observed by SEM. The fractural changes were obvious in the microstructures of LPS after extraction. The outermost layers of LSP showed integrity before extraction (Figure 5a), but it started to become damaged after treating with WW (Figure 5b), in the case of glycerol the damage was obvious (Figure 5c). When treated by UW (Figure 5d), the cell walls were disrupted. Meanwhile, the entire shape and structure remained. The cell walls were completely crushed and underwent internal splitting when treated with UG (Figure 5e), leading to the solvent entering the structure of particles, which was favorable for the extraction efficiency of TPC, thereby, the phenomenon might be due to the cavitation from ultrasound, which explained the highest antioxidant activities treated by UG, resulting in more phenolic compounds released from LSP [13].

## 4. Conclusions

In this research, the extraction of phenolic compounds from LSP assisted by ultrasound coupled with green solvent glycerol was performed, and the yield of phenolic compounds extracted from LSP was dramatically increased in a shorter time compared with the traditional extraction methods. The protocol of RSM based on BBD was used to optimize the extraction yield of TPC, and the results revealed that the highest extraction yield of 92.84 ± 2.13 mg GAE/g was obtained by using ultrasonic power 400 W, the concentration of glycerol of 40%, extraction temperature of 66 °C, ultrasonic time of 44 min and the solvent-to-solid ratio of 55 mL/g. Under these conditions, the antioxidant capacity was evaluated by using DPPH, FRAP, ABTS, and RA assays. Meanwhile, UPLC-Triple-TOF/MS was used to identify the constituents of LSP extract, and quercetin 3-*O*-glucuronide was the predominant compound. Likewise, FTIR indicated the presence of polyphenols and flavonoids, and SEM revealed the difference of microstructure of LSP samples amongst four different extraction procedures, illustrating the mechanism of best extraction efficiency. In summary, ultrasonic-assisted coupled with glycerol is a simple, inexpensive, and efficient alternative compared with conventional extraction technologies, which is more attractive and promising in the food industry to produce safe and natural antioxidants.

## Figures and Tables

**Table 1:** **The** variables levels for the experimental design

Independent Variables	Levels		
	−1	0	1
X_1_: concentration of glycerol (%)	25	35	45
X_2_: ultrasonic time (min)	30	40	50
X_3_: temperature (°C)	50	60	70
X_4_: solvent-to-solid ratio (mL/g)	40	50	60

**Table 2:** The experimental and predicted values of 29 experiments obtained with BBD for TPC.

Run	X_1_: Concentration of Glycerol	X_2_: Ultrasonic Time (min)	X_3_: Temperature (°C)	X_4_: Solvent-to-Solid Ratio	Experimental Values	Predicted Values
1	45.00	40.00	70.00	50.00	89.2097	88.85
2	35.00	30.00	60.00	60.00	82.0877	82.06
3	35.00	40.00	60.00	50.00	88.8395	89.19
4	45.00	40.00	60.00	40.00	82.9761	82.93
5	35.00	30.00	60.00	40.00	80.3109	79.98
6	45.00	40.00	50.00	50.00	78.9191	78.90
7	35.00	40.00	60.00	50.00	89.0616	89.19
8	25.00	40.00	50.00	50.00	72.1821	72.41
9	35.00	50.00	60.00	40.00	82.2654	82.17
10	35.00	50.00	60.00	60.00	86.6185	86.82
11	25.00	40.00	60.00	40.00	78.1788	78.26
12	45.00	50.00	60.00	50.00	87.729	87.52
13	45.00	30.00	60.00	50.00	81.2882	81.72
14	35.00	50.00	50.00	50.00	77.2164	77.45
15	35.00	50.00	70.00	50.00	88.0252	88.47
16	25.00	40.00	70.00	50.00	82.9169	82.81
17	25.00	30.00	60.00	50.00	77.7346	77.78
18	25.00	40.00	60.00	60.00	79.6891	80.02
19	35.00	30.00	70.00	50.00	84.1014	84.15
20	45.00	40.00	60.00	60.00	87.6846	87.89
21	35.00	40.00	60.00	50.00	89.0616	89.19
22	35.00	40.00	70.00	60.00	89.8168	89.53
23	35.00	40.00	50.00	40.00	75.869	76.00
24	35.00	40.00	70.00	40.00	84.3976	84.66
25	25.00	50.00	60.00	50.00	79.5114	78.93
26	35.00	30.00	50.00	50.00	74.9954	74.83
27	35.00	40.00	60.00	50.00	89.2837	89.19
28	35.00	40.00	60.00	50.00	89.7279	89.19
29	35.00	40.00	50.00	60.00	78.2676	77.85

**Table 3:** Analysis of variance for response surface quadratic models.

Source	Sum of Squares	df	Mean Square	*F*-Value	*p*-Value Prob > F
Model	751.08	14	53.65	346.68	<0.0001
X_1_- concentration of glycerol	117.77	1	117.77	761.08	<0.0001
X_2_- ultrasonic time	36.22	1	36.22	234.05	<0.0001
X_3_- temperature	310.27	1	310.27	2004.97	<0.0001
X_4_- solvent-to-solid ratio	33.89	1	33.89	219.01	<0.0001
X_1_X_2_	5.44	1	5.44	35.14	<0.0001
X_1_X_3_	0.05	1	0.049	0.32	0.5813
X_1_X_4_	2.56	1	2.56	16.52	0.0012
X_2_X_3_	0.72	1	0.72	4.68	0.0482
X_2_X_4_	1.66	1	1.66	10.72	0.0055
X_3_X_4_	2.28	1	2.28	14.74	0.0018
X_1_^2^	108.75	1	108.75	702.77	<0.0001
X_2_^2^	84.61	1	84.61	546.74	<0.0001
X_3_^2^	123.16	1	123.16	795.89	<0.0001
X_4_^2^	51.83	1	51.83	334.95	<0.0001
Residual	2.17	14	0.15		
Lack of Fit	1.71	10	0.17	1.51	0.3679
Pure Error	0.45	4	0.11		
Cor Total	753.25	28			
C.V.%	0.47				
Adeq Precision	60.502				
*R* ^2^	0.9971				
Adj *R*^2^	0.9942				
Pred *R*^2^	0.9860				

**Table 4:** Extraction yields of TFC, TCTC, and TTC with four different treatments.

Sample	TFC	TCTC	TTC
	(mg QE/g)	(mg CE/g)	(mg TAE/g)
UG	70.09 ± 3.35a	70.73 ± 4.76a	68.94 ± 1.44b
UW	72.41 ± 3.83a	40.19 ± 0.72c	47.22 ± 0.57c
WG	60.97 ± 2.75b	65.05 ± 2.95b	72.27 ± 1.70a
WW	58.05 ± 7.01b	40.23 ± 0.92c	41.26 ± 0.89d

Data are reported as mean ± SD (*n* = 6). Different letters, such as a, b, c, and d showed significant difference (*p* < 0.05).

## Data Availability

The data presented in this study are available in Supplementary Materials (Figures S1–S9).

## Supplementary Materials

The following are available online at https://www.mdpi.com/2304-8158/10/2/239/s1, Figure S1: The standard curve of Gallic acid for TPC, Figure S2: The standard curve of Quercetin for TFC, Figure S3: The standard curve of Catechin for TCTC, Figure S4: The standard curve of Tannin acid for TTC, Figure S5: The standard curve of Trolox for DPPH, Figure S6: The standard curve of Trolox for FRAP, Figure S7: The standard curve of Trolox for ABTS; Figure S8: The standard curve of Trolox for RA, Figure S9. Basic peek chromatograms of Receptaculum Nelumbinis extract obtained by ultrasound coupled with glycerol.
