# Supplementary material for: Green Extraction of Phenolic Compounds from Lotus Seedpod (Receptaculum Nelumbinis) Assisted by Ultrasound Coupled with Glycerol
Source: Foods. 2021 Jan 25;10(2):239. doi: 10.3390/foods10020239 (PMC7912186; doi:10.3390/foods10020239)
Supplement: Supplementary file 1 [file foods-10-00239-s001.pdf]

Supplementary File:

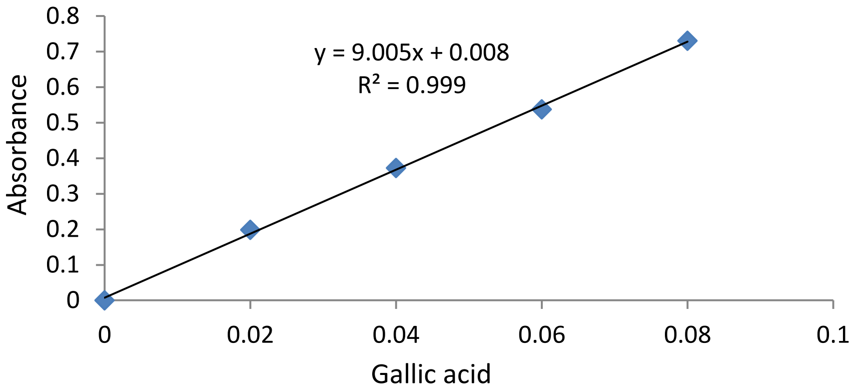

Figure S1. The standard curve of Gallic acid for TPC.

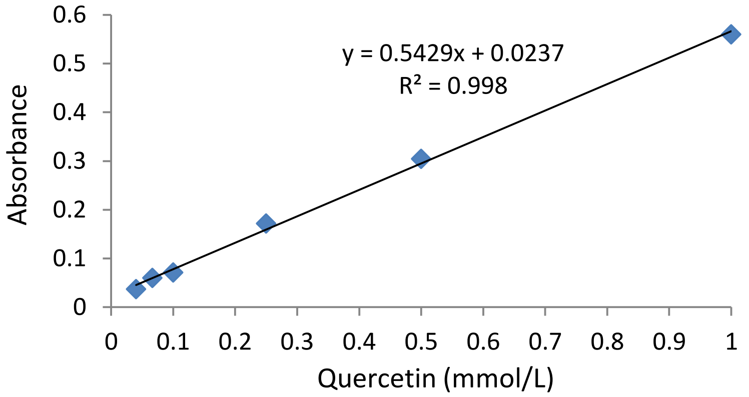

Figure S2. The standard curve of Quercetin for TFC.

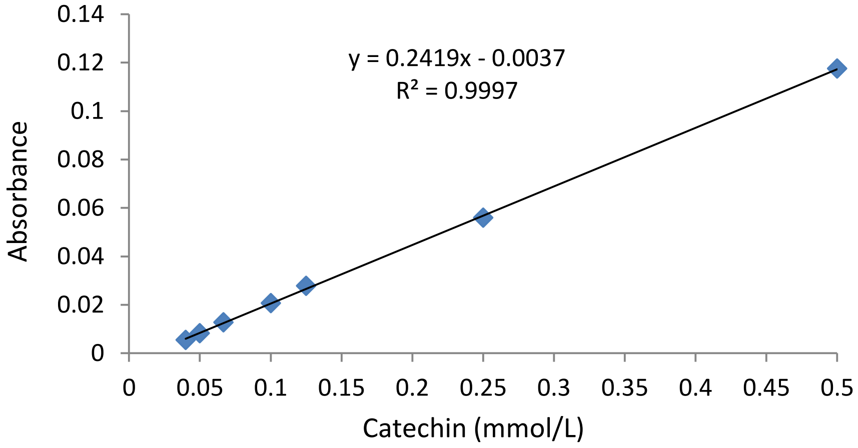

Figure S3. The standard curve of Catechin for TCTC.

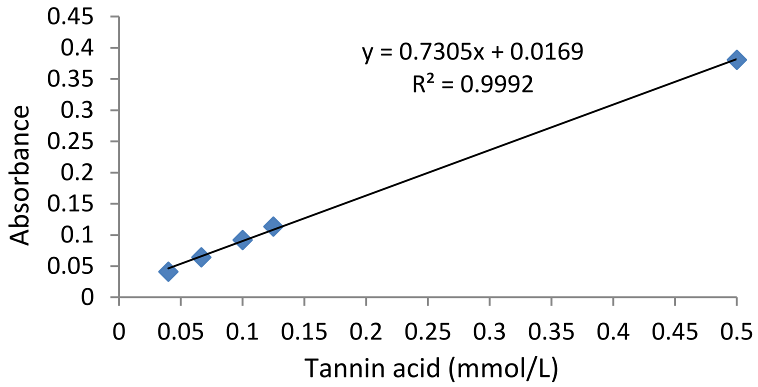

Figure S4. The standard curve of Tannin acid for TTC.

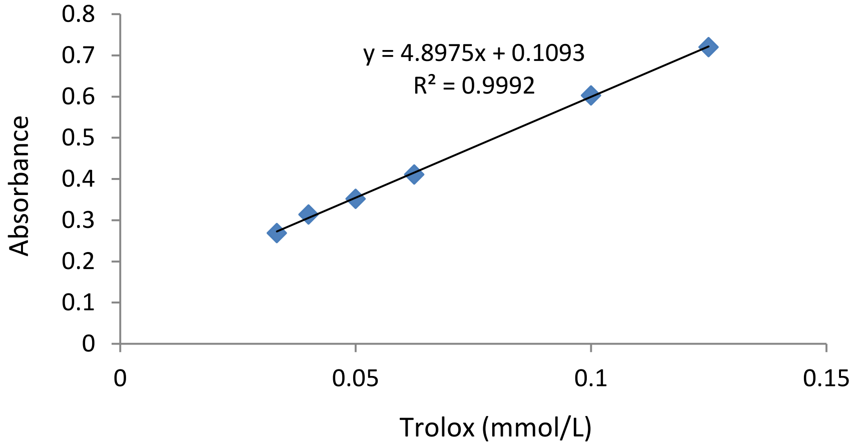

Figure S5. The standard curve of Trolox for DPPH.

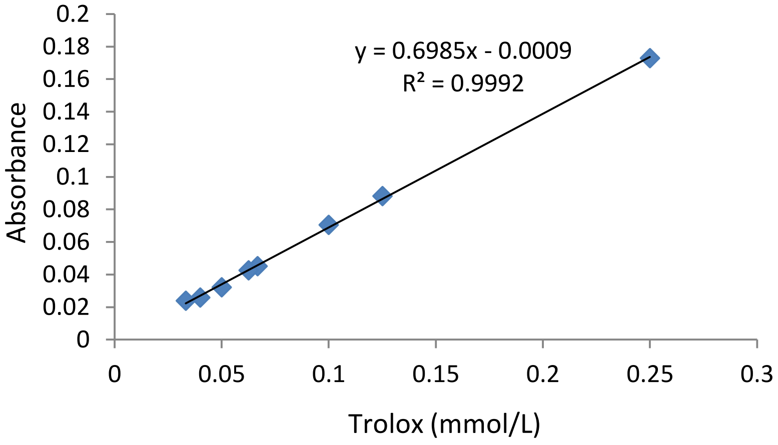

Figure S6. The standard curve of Trolox for FRAP.

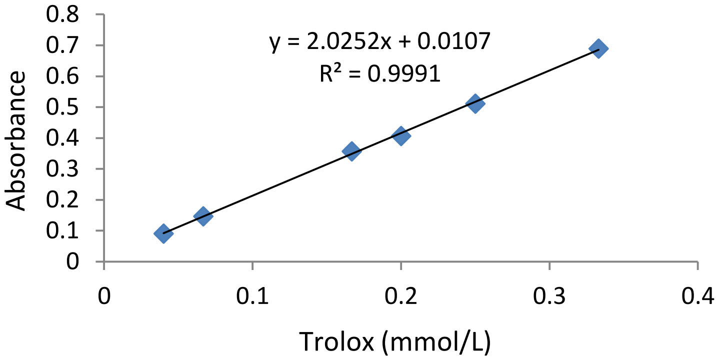

Figure S7. The standard curve of Trolox for ABTS.

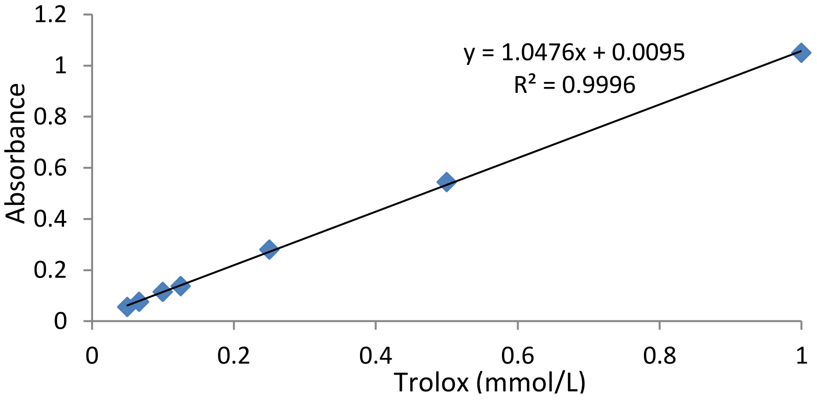

Figure S8. The standard curve of Trolox for RA.

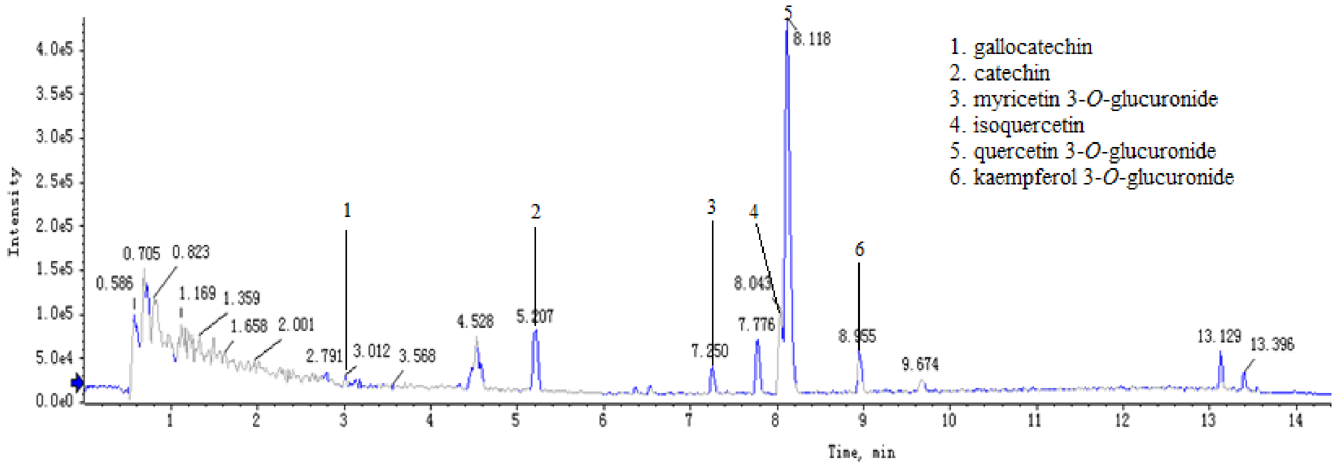

Figure S9. Basic peek chromatograms of Receptaculum Nelumbinis extract obtained by ultrasound coupled with glycerol.
